# Drug Resistance of *Mycobacterium tuberculosis* Complex in a Rural Setting, Angola

**DOI:** 10.3201/eid2403.171562

**Published:** 2018-03

**Authors:** Ariadna Rando-Segura, María Luisa Aznar, María Milagros Moreno, Mateu Espasa, Elena Sulleiro, Cristina Bocanegra, Eva Gil, Arlete N.E. Eugénio, Carlos Escartin, Adriano Zacarias, Josep Vegue, Domingos Katimba, María Carmen Vivas, Estevao Gabriel, María Concepción Marina, Jacobo Mendioroz, María Teresa López, Tomas Pumarola, Israel Molina, María Teresa Tórtola

**Affiliations:** Vall d’Hebron University Hospital, PROSICS Barcelona, Universitat Autònoma de Barcelona, Barcelona, Spain (A. Rando-Segura, M.L. Aznar, M. Espasa, E. Sulleiro, C. Bocanegra, E. Gil, C. Escartin, J. Vegue, M.C. Vivas, M.C. Marina, J. Mendioroz, T. Pumarola, I. Molina, M.T. Tórtola);; Hospital Nossa Senhora da Paz, Cubal, Angola (M.L. Aznar, M.M. Moreno, C. Bocanegra, E. Gil, A.N.E. Eugénio, A. Zacarias, D. Katimba, E. Gabriel, M.T. López)

**Keywords:** Angola, Cubal, GenoType MTBDRplus, drug susceptibility testing, resistance, multidrug resistance, multidrug resistant, MDR, extensive drug resistance, extensive drug resistant, XDR, Mycobacterium tuberculosis, TB, tuberculosis and other mycobacteria, bacteria, bacterial infection, antimicrobial resistance

## Abstract

We found high prevalence rates of multidrug-resistant tuberculosis among retreatment patients (71.1%) and persons with new cases (8.0%) in Angola. These findings are of concern but should be interpreted with caution. A national drug-resistance survey is urgently needed to determine the actual prevalence of multidrug-resistant tuberculosis in Angola.

Angola is among the 30 countries with the highest incidence of tuberculosis (TB) and multidrug-resistant (MDR) TB worldwide (*1*). However, drug-resistance prevalence is unknown in the absence of a national survey or laboratory drug-resistance surveillance systems (*1*). The objectives of our study were to determine the proportion of TB drug resistance in isolates from pulmonary TB patients and describe molecular mechanisms accounting for drug resistance in these isolates.

## The Study

We conducted a survey during April 2014–July 2015 at the Nossa Senhora da Paz Hospital (HNSP), a reference center for the diagnosis and treatment of TB in the town of Cubal, Benguela Province, Angola. Patients >16 years of age with a diagnosis of pulmonary TB (i.e., patients with clinical symptoms and a positive smear result) and those infected with HIV who had suggestive clinical signs of pulmonary TB but negative sputum samples for acid-fast bacilli were eligible for enrollment in the study. We collected data on age, sex, HIV status, and any previous TB treatment.

Before the start of treatment, we collected sputum specimens from all case-patients and provided them to the Mycobacteriology Unit (a World Health Organization Supranational TB Reference Laboratory) at Vall d’Hebron University Hospital in Barcelona, Spain, for culture and drug-susceptibility testing. Positive cultures were tested by using GenoType MTBDR*plus* 2.0 (Hain Lifescience GmbH, Nehren, Germany). Isolates identified as *Mycobacterium tuberculosis* complex (MTBC) underwent drug-susceptibility testing with BD-MGIT-960 SIRE and PZA kits (Becton Dickinson Diagnostic Systems, Sparks, MD, USA). Isolates that were resistant to >1 drug were subjected to drug-susceptibility testing for second-line TB drugs by using the BD-MGIT-960 SIRE system.

We performed statistical analysis by using Stata 12 (StataCorp LLC, College Station, TX, USA). We considered a p value <0.05 to be statistically significant. We calculated the percentage of patients with resistance patterns to first- and second-line TB drugs on the basis of total number of cases and the total number of MDR TB cases, respectively.

We included 422 cases; 44 were excluded because sputum specimen was not obtained (Technical Appendix Figure). Of these cases, we classified 311 as new and the remaining 111 as retreatment cases. We isolated MTBC in 225 of the new cases. We observed no difference in the distribution of age, sex, or HIV status between case-patients with suspected or confirmed TB disease among the new cases. We isolated MTBC in 83 of the retreatment cases. We found case-patients in whom MTBC was not isolated were more frequently HIV-positive (14.3% compared with 4.8% in whom TB was confirmed; p = 0.09). We observed no difference in sociodemographic characteristics between patients with new and retreatment culture-positive cases (Technical Appendix Table 1).

Eighteen (8.0%) of the 225 MTBC isolates from new cases demonstrated multidrug resistance. Other combinations of drug resistance were identified in 40 (17.8%) of new cases. The incidence of primary resistance was as follows: isoniazid, 47 cases (20.9%); streptomycin, 25 cases (11.1%); rifampin, 20 cases (8.9%); pyrazinamide, 13 cases, (5.8%); and ethambutol, 10 cases (4.4%) (Table 1). No isolates showed extensively drug-resistant TB (Technical Appendix Table 2). 

**Table 1:** Resistance to first-line antituberculosis drugs among *Mycobacterium tuberculosis* complex isolates, Cubal, Angola, April 2014–July 2015*

Phenotypic drug susceptibility	Isolates from new cases, n = 225			Isolates from retreatment cases, n = 83	
	No.	% (95 CI)		No.	% (95 CI)
Susceptible to all 5 first-line drugs	167	74.2 (68.1–79.5)		14	16.9 (10.3–26.3)
Resistance to any drug	58	25.8 (20.5–31.9)		69	83.1 (73.7–89.7)
Any resistance to the following					
INH	47	20.9 (16.1–26.7)		66	79.5 (69.6–86.8)
RIF	20	8.9 (5.8–13.3)		61	73.5 (63.1–81.8)
STM	25	11.1 (7.6–15.9)		42	50.6 (40.1–61.1)
EMB	10	4.4 (2.4–8.0)		32	38.6 (28.8–49.3)
PZA	13	5.8 (3.4–9.6)		37	44.6 (34.4–55.3)
Overall monodrug resistance	31	13.8 (9.9–18.9)		7	8.4 (4.1–16.4)
INH only	21	9.3 (6.2–13.8)		4	4.8 (1.9–11.7)
RIF only	1	0.4 (0.1–2.5)		2	2.4 (0.7–8.4)
STM only	8	3.6 (1.8–6.9)		1	1.2 (0.2–6.5)
PZA only	1	0.4 (0.1–2.5)		0	0.0 (0.0–4.4)†
Overall multidrug resistance	18	8.0 (5.1–12.3)		59	71.1 (60.6–79.7)
INH + RIF	4	1.8 (0.7–4.5)		12	14.5 (8.5–23.6)
INH + RIF + STM	2	0.9 (0.2–3.2)		5	6.0 (2.6–13.3)
INH + RIF + EMB	0	0.0 (0.0–1.7)†		3	3.6 (1.2–10.1)
INH + RIF + PZA	2	0.9 (0.2–3.2)		2	2.4 (0.7–8.4)
INH + RIF + STM + EMB	3	1.3 (0.5–3.8)		4	4.8 (1.9–11.7)
INH + RIF + STM + PZA	1	0.4 (0.1–2.5)		9	10.8 (5.8–19.3)
INH + RIF + EMB + PZA	1	0.4 (0.1–2.5)		4	4.8 (1.9–11.7)
INH + RIF + STM + EMB + PZA	5	2.2 (1.0–5.1)		20	24.1 (16.2–34.3)
Overall polydrug resistance	9	4.0 (2.1–7.4)		3	3.6 (1.2–10.1)
INH + STM	5	2.2 (1.0–5.1)		0	0.0 (0.0–4.4)†
INH + EMB	1	0.4 (0.1–2.5)		0	0.0 (0.0–4.4)†
INH + PZA	2	0.9 (0.2–3.2)		0	0.0 (0.0–4.4)†
INH + STM + EMB	0	0.0 (0.0–1.7)†		1	1.2 (0.2–6.5)
INH + STM + PZA	0	0.0 (0.0–1.7)†		2	2.4 (0.7–8.4)
RIF + STM + PZA	1	0.4 (0.1–2.5)		0	0.0 (0.0–4.4)†


*EMB, ethambutol; INH, isoniazid; PZA, pyrazinamide; RIF, rifampin; STM, streptomycin. †1-sided, 97.5% CI.

Among the 47 isoniazid-resistant isolates, *katG* mutations occurred in 26 (55.3%) and *inhA* mutations in 2 (4.3%); the remaining 19 isolates (40.4%) were classified as susceptible (Table 2). Among the 20 rifampin-resistant isolates, *rpoB* mutations occurred in 19 (95.0%), and 1 (5.0%) was classified as susceptible. Mutations detected included S531L (12 cases, 60.0%); D516V (4 cases, 20.0%); and H526Y (2 cases, 10.0%) (Table 2).

**Table 2:** Distribution of gene mutations associated with INH and RIF resistance, Cubal, Angola, April 2014–July 2015*

Phenotypic resistance	GenoType MTBDR*plus*			Isolates from new cases	Isolates from retreatment cases
	*katG*	*inhA*	*rpoB*		
INH, n = 113				47	66
	∆wt, S315T1	–		23	42
	∆wt	–		3	5
	–	∆wt1, C15T		2	2
	–	∆wt1		0	1
	**–**	C15T		0	1
	–	–		19	15
RIF, n = 81				20	61
			–	1	3
			Δwt2	0	2
			Δwt2,3,4, D516V	1	8
			Δwt3,4, D516V	3	3
			Δwt4,5	0	1
			Δwt7	0	1
			Δwt7, H526Y	2	0
			Δwt7, H526D	0	2
			Δwt7,8, H526D	0	1
			Δwt8	1	3
			Δwt8, S531L	12	37

*INH, isoniazid; RIF, rifampin; –, no mutation inside region.

Fifty-nine (71.1%) of the 83 MTBC isolates from retreatment case-patients demonstrated multidrug resistance, and 33.9% of these case-patients had isolates that were resistant to all first-line drugs. Other combinations of drug resistance were identified in 10 case-patients (12.0%) (Table 1). No case-patients had extensively drug-resistant TB (Technical Appendix Table 2).

Among the 66 isoniazid-resistant isolates, *katG* mutations occurred in 47 (71.2%) and *inhA* mutations in 4 (6.1%); the remaining 15 (22.7%) isolates were classified as susceptible (Table 2). Among the 61 rifampin-resistant isolates, *rpoB* mutations occurred in 58 (95.1%), and the remaining 3 (4.9%) were classified as susceptible. Mutations detected included S531L (37 cases, 60.7%), D516V (11 cases, 18.5%), and H526D (3 cases, 3.7%) (Table 2).

## Conclusions

We found a high prevalence of MDR TB among retreatment (71.1%) and new (8.0%) cases. These rates are >4 times the estimated prevalence of MDR TB for Angola (21% for retreatment cases, 2.8% for new cases) (*1*). The rates we describe represent the highest rates of MDR TB reported in sub-Saharan Africa (*2**,**3*); not even South Africa has reported a higher prevalence of MDR TB (*4*). 

Our findings are part of a larger project to reinforce the capacities of the diagnostic laboratory by incorporation of the Xpert MTB/RIF test (Cepheid, Maurens-Scopont, France) (*5*). At the beginning of the project, none of the 18 provinces in Angola had access to the test; moreover, Nossa Senhora da Paz Hospital is a reference center for the diagnosis and treatment of TB, and these 2 factors might have generated a pull effect in more severe cases. Patients in the study might have largely consisted of TB patients referred because of poor treatment response or availability of second-line treatment, thus overrepresenting patients with resistance patterns, particularly among retreatment patients. This suggestion is supported by the high proportion of retreatment patients in the eligible study population (26.3% where the expected population proportion would be 10%–15%) and the extremely high prevalence of MDR TB in this group (in particular compared with new patients). Also, for new patients, such selection bias might have occurred, for example, because TB patients who were contacts of known or suspected MDR TB patients were preferentially referred to this facility.

Regarding associated mutations, previous studies have shown that ≈95% of resistance mutations to rifampin are associated with the *rpoB* gene mutations, which cluster mainly in the region of codon 507–533. In our study, the distribution of gene mutations among rifampin-resistant isolates was 60.4% Ser531Leu, 18.5% Asp516Val, 3.7% His526Asp, and 2.5% His526Tyr; in 9.9% of cases, the mutation was detected by the absence of the wild-type hybridization signal. This distribution is different from that previously reported, reflecting different distribution of gene mutations associated with rifampin resistance in different geographic locations (*6*) or different levels of maturation of the MDR TB epidemic. In areas with high MDR TB prevalence and a high proportion of MDR TB cases attributed to transmission, mutations that confer resistance without loss of reproductive fitness will be selected out (*7**,**8*).

Whereas 40%–95% of isoniazid-resistant isolates are defined as having high-level drug resistance because of *katG* gene mutations, 75%–90% of which are recognized as mutations in the 315 codon of the *katG* gene, in our study, 57.5% of isoniazid-resistant isolates were associated with mutations in the 315 codon of the *katG* gene. Approximately 8%–43% of isoniazid-resistant isolates are defined as having low-level drug resistance because of mutations in the promoter region of *inhA*. In our study, this proportion was 5.3%. Furthermore, 10%–25% of isoniazid-resistant isolates are thought to have mutations outside the *katG* and *inhA* loci (*9**–**11*).

Although the high prevalence rate of MDR TB we observed among the patients in our study is of concern, these findings should be interpreted with caution. A national drug-resistance survey is urgently needed to assess the actual prevalence of MDR TB in Angola.

Technical Appendix Sociodemographic characteristics of study participants, patterns of resistance to second-line antituberculosis drugs among multidrug-resistant *Mycobacterium tuberculosis* complex isolates, and schematic overview of the study, Cubal, Angola, April 2014–July 2015.
