# Supplementary material for: Drug Resistance of Mycobacterium tuberculosis Complex in a Rural Setting, Angola
Source: Emerg Infect Dis. 2018 Mar;24(3):569–72. doi: 10.3201/eid2403.171562 (PMC5823346; doi:10.3201/eid2403.171562)
Supplement: Technical Appendix — Sociodemographic characteristics of study participants, patterns of resistance to second-line antituberculosis drugs among multidrug-resistant Mycobacterium tuberculosis complex isolates, and schematic overview of the study, Cubal, Angola, April 2014–July 2015. [file 17-1562-Techapp-s1.pdf]

Article DOI: https://doi.org/10.3201/eid2403.171562

Drug Resistance of *Mycobacterium tuberculosis* Complex in a Rural Setting, Angola

Technical Appendix

Technical Appendix Table 1. Sociodemographic characteristics of study participants diagnosed with pulmonary TB in Cubal, Angola, April 2014–July 2015

		New cases (n = 311)		Retreatment cases (n = 111)	
Characteristic		Confirmed TB (n = 225)	Suspected TB (n = 86)	Confirmed TB (n = 83)	Suspected TB (n = 28)
Age	Median (IRC)	28.5 (IRC: 23.5–38.5)	32.5 (IRC: 23.5–43.3)	30.5 (IRC: 25.0–35.5)	29.5 (IRC: 24.8–42.0)
Sex	Male	145 (64.4%)	53 (61.6%)	47 (56.6%)	16 (57.1%)
	Female	80 (35.6%)	33 (38.4%)	36 (43.4%)	12 (42.9%)
HIV	Positive	12 (5.3%)	8 (9.3%)	4 (4.8%)	4 (14.3%)
	Negative	210 (93.3%)	77 (89.5%)	79 (95.2%)	24 (85.7%)
AFB/ smear	Negative	6 (2.7%)	27 (31.4%)	5 (6.0%)	8 (28.6%)
	Low burden	25 (11.1%)	28 (32.6%)	7 (8.4%)	8 (28.6%)
	High burden	194 (86.2%)	31 (36.0%)	71 (85.5%)	12 (42.9%)

Abbreviations: AFB = acid fast bacilli. Definitions: “Low burden” were defined as patients with 1–9 AFB / 100 fields or 10–99 AFB / 100 fields. “High burden” were defined as patients with 1–10 AFB / field or >10 AFB / field.

Technical Appendix Table 2. Resistance to second-line anti-tuberculosis drugs among multidrug-resistant *Mycobacterium tuberculosis* complex isolates, Cubal, Angola, April 2014–July 2015

	Isolates from new cases (n = 18)		Isolates from retreatment cases (n = 59)	
Phenotypic drug susceptibility	No.	% (95 CI)	No.	% (95 CI)
Susceptible	16	83.3 (60.8 – 94.2)	50	84.7 (73.5 – 91.8)
Any resistance to FQ †	0	0.0 (0.0 – 17.6)*	0	0.0 (0.0 – 6.1)*
Any resistance to 2LI ‡	2	16.7 (5.8 – 39.2)	9	15.3 (8.2 – 26.5)
Any resistance to both FQ † and 2LI ‡ (XDR-TB)	0	0.0 (0.0 – 17.6)*	0	0.0 (0.0 – 6.1)*

Abbreviations: FQ = fluoroquinolones; 2LI = second line injectable drugs; XDR = extensively drug resistant. (†) Drugs tested: ofloxacin at 2.0 µg/ml and moxifloxacin at 0.25 µg/ml. (‡) Drugs tested: amikacin at 1.0 µg/ml and capreomycin at 2.5 µg/ml. (*) one-sided, 97.5% confidence Interval.

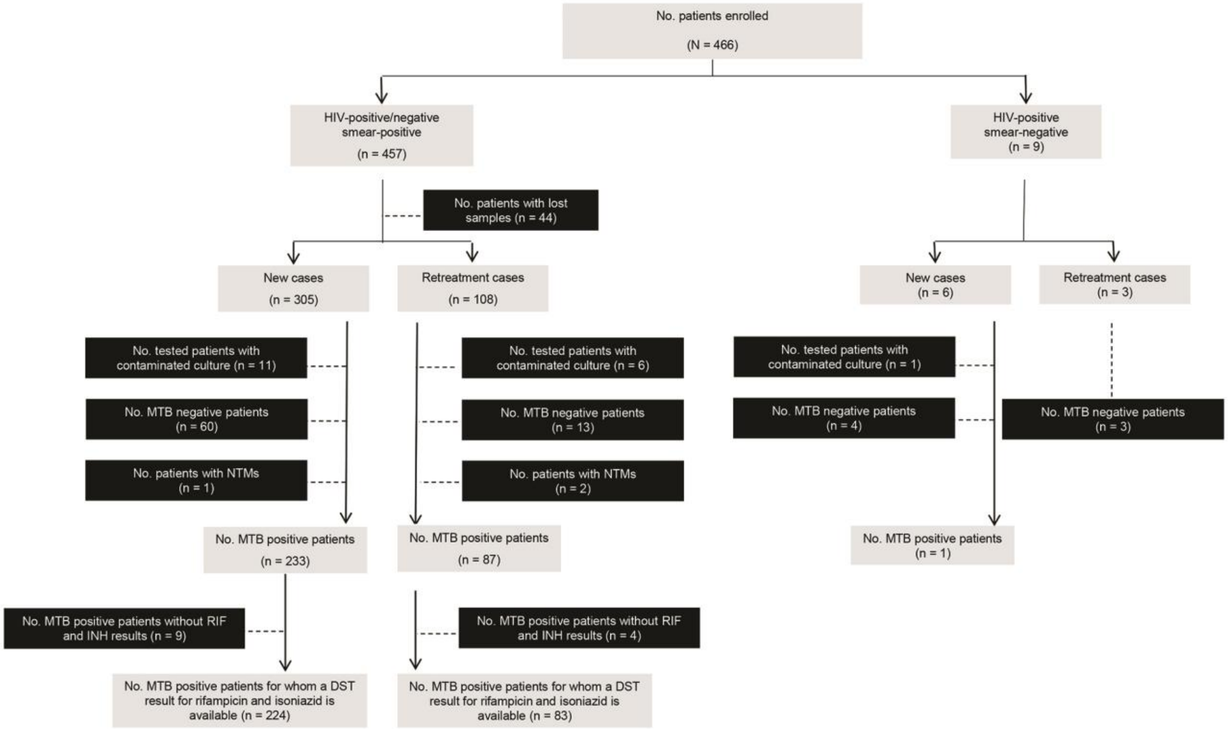

Technical Appendix Figure. Schematic overview of the study, Cubal, Angola, April 2014–July 2015.
